# Editorial: Plasticity of the haematopoietic niche: from embryonic development to aging and disease

**DOI:** 10.3389/fmolb.2025.1683902

**Published:** 2025-08-28

**Authors:** Rio Sugimura, Antonella Fidanza, Emanuele Azzoni, Giovanni Canu

**Affiliations:** 1 Li Ka Shing Faculty of Medicine, School of Biomedical Sciences, University of Hong Kong, Hong Kong, Hong Kong SAR, China; 2 Centre for Translational Stem Cell Biology, Hong Kong, Hong Kong SAR, China; 3 Centre for Regenerative Medicine, Institute for Regeneration and Repair, Edinburgh Medical School, Biomedical Sciences, University of Edinburgh, Edinburgh, United Kingdom; 4 Edinburgh Medical School, Biomedical Sciences, University of Edinburgh, Edinburgh, United Kingdom; 5 School of Medicine and Surgery, University of Milano-Bicocca, Monza, Italy; 6 Fondazione IRCCS San Gerardo dei Tintori, Monza, Italy; 7 UCL Institute of Ophthalmology, University College London, London, United Kingdom

**Keywords:** erythro-myeloid progenitors, fetal liver, bone marrow, hematopoietic stem cells (HSCs), hematopoietic niche, embryonic development, hematopoietic development, yolk sac (YS)

**FIGURE 1:**
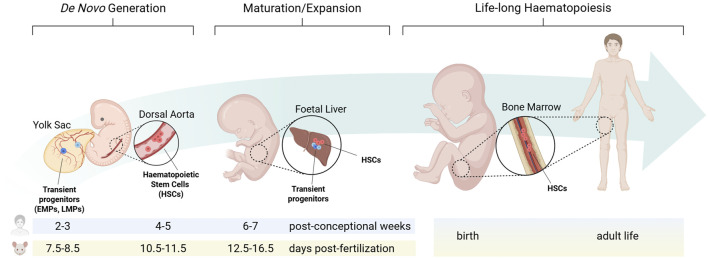
The haematopoietic niche from embryonic development to adult homeostasis. During embryonic development, distinct haematopoietic progenitors arise from separate niches at defined times. Each population transiently migrates to the foetal liver, where they sustain foetal blood and immune cell formation, before haematopoietic stem cells colonise the bone marrow during late gestation to establish adult long-term haematopoiesis. EMPs: erythro-myeloid progenitors; LMPs: lympho-myeloid progenitors; HSCs: haematopoietic stem cells. Created in BioRender. Canu et al. (2025) https://BioRender.com/vantnnf.

The site of origin for new haematopoietic cells and the niche that sustains their self-renewal and differentiation undergo significant changes from embryonic development to adult homeostasis (Figure 1).

In the developing embryo, haematopoiesis proceeds through three temporally and spatially overlapping waves (Canu and Ruhrberg, 2021; Barone et al., 2022). Primitive haematopoiesis, the first wave of blood cell formation, originates in the mouse yolk sac from embryonic day (E) 7.0, equivalent to 2-3 post-conception weeks in human, when extra-embryonic mesoderm differentiates into haemato-vascular progenitors gradually assembling into blood islands that generate nucleated erythrocytes, megakaryocytes and macrophages (Ferkowicz and Yoder, 2005). Pro-definitive haematopoiesis, the second haematopoietic wave, also arises in the yolk sac. During this phase, haemogenic endothelial cells undergo a trans-differentiation process known as endothelial-to-haematopoietic transition (EHT), producing clusters of haematopoietic progenitors that consist primarily of erythro-myeloid progenitors (EMPs), along with lympho-myeloid progenitors (LMPs) (Frame et al., 2013; Hoeffel et al., 2015). In the mouse, EMPs arise from E8.25, while LMPs from E9.5 (Böiers et al., 2013). With the establishment of the blood circulation by E10.5, these progenitors colonise the foetal liver, from where they sustain haematopoiesis until birth. Furthermore, it was recently shown in the mouse that a distinct wave of haematopoietic progenitors akin to EMPs may arise from paraxial mesoderm, transiently seeding the foetal liver by E12.5 (Canu et al., 2024). Finally, definitive haematopoiesis, the third haematopoietic wave, originates intra-embryonically from the dorsal aorta, where at E10.5 in the mouse, and 4-5 post-conception weeks in human, EHT generate haematopoietic stem cells (HSCs) (Ivanovs et al., 2017; Canu and Ruhrberg, 2021; Barone et al., 2022). Understanding the developing haematopoietic microenvironments is increasingly recognised as critical for producing haematopoietic stem/progenitor cells (HSPCs) *in vitro* (Sugimura et al., 2017; Canu et al., 2020; Ding et al., 2025) and for elucidating childhood leukaemias, many of which are thought to have a foetal origin (Cazzola et al., 2021).

In their comprehensive review, Sánchez-Lanzas et al. describe the HSC journey across multiple niches from embryogenesis to adult life. They first examine HSCs emergence from the dorsal aorta, focusing on the complex network of finely tuned signalling pathways controlling HSC specification. Subsequently, HSCs migrate to the foetal liver, which has been classically considered an expansion niche for recently born HSCs. The authors cite recent evidence challenging this dogma to support an additional role for the foetal liver in HSC maturation, and examine hepatic cell types that may contribute to this dual maturation/expansion function. Next, the authors discuss HSC migration to the bone marrow during late gestation and how this environment changes after birth, triggering HSC transition into a quiescent state. Importantly, this review highlights key but often underestimated differences between the foetal, perinatal and adult bone marrow niche.

Concomitant with HSC migration, blood cell production also shifts from the liver to the bone marrow. However, the timing of this process has been poorly characterised in human. Combining transcriptomics and histological analyses, Janovska et al. analyse liver samples from 25 premature newborns to compare the influence of gestational vs. postnatal age on the perinatal shift from hepatic to bone marrow haematopoiesis. The findings confirmed a progressive decline in total haematopoietic activity in the liver during the third trimester. Interestingly, erythropoiesis decreases after birth significantly faster than granulopoiesis. Specifically, red blood cell production in the liver sharply drops within the first few days of life independently of gestational age, potentially reflecting physiological adaptation to a postnatal oxygen-rich environment. In contrast, liver granulopoiesis displays a slower decline.

Much of our knowledge from the haematopoietic niche derives from post-mortem tissues, limiting our capability to directly study cell and molecular mechanisms. To overcome this limitation, Ventura et al. used a human induced pluripotent stem cell (hiPSC) system to reproduce *in vitro* the erythroblastic island niche within the bone marrow, where a specialised population of macrophages support red blood cell production throughout adult life. The authors demonstrated that hiPSC-derived macrophages could be programmed towards an erythroblastic island phenotype by inducing expression of the erythroid transcription factor KLF1. Furthermore, they used a proteomic approach to characterise changes in KLF1-induced macrophages, suggesting that the erythropoiesis-supportive role may be mediated via production of extracellular vesicles.

In recent years, the use of single-cell transcriptomics has revolutionised our understanding of the haematopoietic niche. Integrating multiple published datasets, Feng et al. built five comprehensive single-cell atlases from key haematopoietic microenvironments: the dorsal aorta, foetal liver, foetal bone marrow, young adult bone marrow and aged adult bone marrow. These datasets collectively encompass over half a million cells annotated into 26 distinct cell types, including various haematopoietic, endothelial and niche-supportive stromal cell populations. Overall, this work provides a high-resolution view of how cellular composition and intercellular signalling evolve in HSC niches across life stages. The ability of the bone marrow to sustain haematopoietic homeostasis declines with aging, possibly due to niche alterations. The authors showed that such alterations include gene expression changes associated with elevated inflammatory signalling and decreased immune activity, iron metabolism, ribosomal function and stress response, overall leading to progressive decline in HSC function and mobilisation capacity.

In addition to an aging niche, the tumour microenvironment can also profoundly influence haematopoietic cells, including neutrophils, macrophages, dendritic cells and progenitors, shaping their activation state, immune behaviour, and ultimately contributing to disease progression and treatment resistance. Zhou et al. used single-cell transcriptomic profiling in gastric cancer patients receiving PD-1 antibody therapy to highlight how specific subclusters of circulating neutrophils become highly activated post-treatment and establish reciprocal interactions with malignant epithelial cells. Thus, epithelial cells secrete chemokines promoting neutrophil recruitment and activation at the tumour site; conversely, neutrophils activate pathways implicated in metastasis and immune evasion. Complementing these findings, Zheng et al. combined bulk and single-cell RNA sequencing to analyse hepatocellular carcinoma samples and define tumour-specific marker genes. Using this approach, they identified a pro-tumour prognostic signature that correlated with increased angiogenesis, infiltration of dendritic cells, macrophages and regulatory T cells, activation of immunosuppressive pathways, and overall associated with poorer survival. Both studies underscore that immune cells are integral components of tumour microenvironments. Their recruitment, activation and reciprocal signalling with tumour cells not only promote tumour progression but also serve as prognostic biomarkers and potential therapeutic targets.

In conclusion, this Research Topic highlights the diversity of haematopoietic niches from embryonic development to adult homeostasis, aging and disease. Recent advances using animal and *in vitro* models, human tissues across life stages, and novel transcriptomic and proteomic technologies, are shedding new light on cell and molecular mechanisms regulating distinct niches.

Overall, haematopoiesis appears to be, now more than ever, a tightly orchestrated yet highly plastic process, controlled by a microenvironment that undergoes life-long remodelling. Decoding such niche plasticity may represent the key step towards developing new and more effective treatments for haematological diseases, including reliable expansion of donor HSCs *in vitro* and their production *de novo* from hiPSCs as a source of transplantable cells.
